# The diagnostic performance of human urinary dipsticks to estimate urine pH, specific gravity (SpG), and protein in horses: are they reliable?

**DOI:** 10.1186/s12917-019-1998-2

**Published:** 2019-07-12

**Authors:** Fatemeh Hekmatynia, Neda Eskandarzadeh, Masoud Imani, Mahdieh Rezaei, Mohamad Zamani-Ahmadmahmudi

**Affiliations:** 1 Department of Clinical Science, Faculty of Veterinary Medicine, Shahid Bahonar University of Kerman, P.O Box: 76169133, Kerman, Iran; 2 Department of Basic Science, Faculty of Veterinary Medicine, Shahid Bahonar University of Kerman, Kerman, Iran

**Keywords:** Horse, Human urinary dipstick, pH, Protein, Specific gravity

## Abstract

**Background:**

Urinalysis is a critical diagnostic test which is performed in routine veterinary medicine practice. In this diagnostic test, semiquantitative measurement of urine biochemical substances is carried out using urinary dipstick. In the current study, we evaluated the diagnostic performance of human urinary dipsticks to estimate pH, specific gravity (SpG), and protein in 80 urine specimens collected from horses. These parameters were measured using two commercial human dipsticks (KP and MN in abbreviation) and quantitative reference methods. The reference methods for pH, SpG, and protein were pH meter, handheld refractometer, and pyrogallol red method, respectively. The correlation between the semiquantitative dipstick analysis and quantitative reference methods was determined using Spearman’s rank correlation coefficient.

**Results:**

In general, our results revealed that the both human urinary dipsticks are unreliable tests for urinary pH, SpG, and protein content in horses. The analysis indicated that there was a poor correlation between the urine dipsticks and reference method (KP: r_S_ = 0.534 and MN: r_s_ = 0.485, *Ps* < 0.001) for protein. Additionally, there was a weak correlation between the results of pH measured using the urine dipsticks and reference method (KP: r_S_ = 0.445 and MN: r_s_ = 0.370, *Ps* < 0.001). Similar findings were obtained for SpG (KP: r_S_ = 0.285, MN: r_s_ = 0.338, *Ps* < 0.001). The estimation of proteinuria using the human dipsticks in horses lacked specificity, as many false positive protein results were obtained.

**Conclusion:**

We observed that the human commercial urinary dipsticks used in this study were not reliable to correctly estimate urine protein, SpG, and pH in horses.

## Background

Urinalysis plays a critical role in early diagnosis of renal disorders and lower urinary tract diseases. Although histopathologic evaluation is considered as a gold-standard test for the definitive diagnosis of many renal diseases, it is invasive, expensive, and time-consuming [1–3]. Hence, a method is required that does not suffer such limitations, is easy to use, and provides us with some rapid and reliable findings. Similar to humans, urinalysis using urine dipstick is routinely performed in veterinary practice. In various studies, the performance of human dipsticks was explored in domestic animals including dogs, cats, cattle, and sheep [1, 4–11]. These dipsticks are predominantly designed for usage in humans and their results in animal samples should be confirmed and interpreted with caution. For instance, previous investigations revealed that SpG and leukocyte count measured using human dipsticks in animal urine were not reliable [12, 13]. Furthermore, a high number of false positive protein results were obtained while assessing human dipsticks in urine samples of cattle, dogs, and cats [4, 5].

To the best of our knowledge, there is no study on the validation of human urinary dipsticks for horse urine. Hence, we selected three important urinary parameters, namely pH, SpG, and protein, and then evaluated them using two human dipsticks (Medi-Test Combi 11 [Machery-Nagel, Germany] and Kimia-Pajouhan [Iran]) along with quantitative reference methods. The results of the semiquantitative method were checked using the reference methods. From now on, we call these dipsticks “MN and KP”, respectively.

## Results

Calcium carbonate and calcium oxalate crystals were observed in 80 (100%) and 7 (8.7%) unstained urine sediments, respectively. Furthermore, granular casts were detected in two samples (2.5%). Our analyses focused on three factors including urinary pH, SpG, and protein.

For all the parameters, inter-rater agreements between the two observers were very good for both dipsticks (*ks* > 0.83) (Table 1). The inter-rater agreement between the two commercial dipsticks was moderate for protein (*k* = 0.60). There was a poor correlation between the urine dipsticks and reference method for protein (KP: r_S_ = 0.534, MN: r_s_ = 0.485, *P*s < 0.001) (Table 1). Although both dipsticks had high sensitivity to detect proteinuria, PPVs of both dipsticks were very low (KP = 4%, MN = 10%), suggesting that a positive result could not be reliable (Table 2). In other words, both dipsticks recorded many false positive results.

**Table 1:** The correlation of two human commercial urinary dipsticks with the corresponding reference methods to measure protein, pH and specific gravity (SpG). Inter-rater agreement between two observers and two dipsticks were also provided

Parameter	Inter-observer agreement		Inter-rater (dipsticks) agreement	Spearman’s rank correlation coefficients between human dipsticks and the reference method	
	KP	MN		KP	MN
Protein	0.87	0.84	0.60	0.534	0.485
pH	0.87	0.85	0.65	0.445	0.370
SpG	0.86	0.86	0.60	0.285	0.338

**Table 2:** Frequency of various semi-quantitative protein measurements performed using two human urinary dipsticks (i.e KP and MN). Furthermore parameters regarding clinical performance of these dipsticks were also provided

	Frequency of various semi-quantitative measurements (mg/dl)				Clinical performance parameters (%)(95% CI^a^)			
	0	30	100	500	Sensitivity	Specificity	PPV	NPV
KP	51 (63.7%)	15 (18.7%)	9 (11.2%)	5 (6.2%)	100 (88–100)	56 (45–60)	4 (2–5)	100 (89–100)
MN	70 (87.5%)	7 (8.5%)	2 (2.5%)	1 (1.2%)	100 (90–100)	86 (73–92)	10 (6–13)	100 (85–100)

^a^confidence interval

**Fig. 1:**
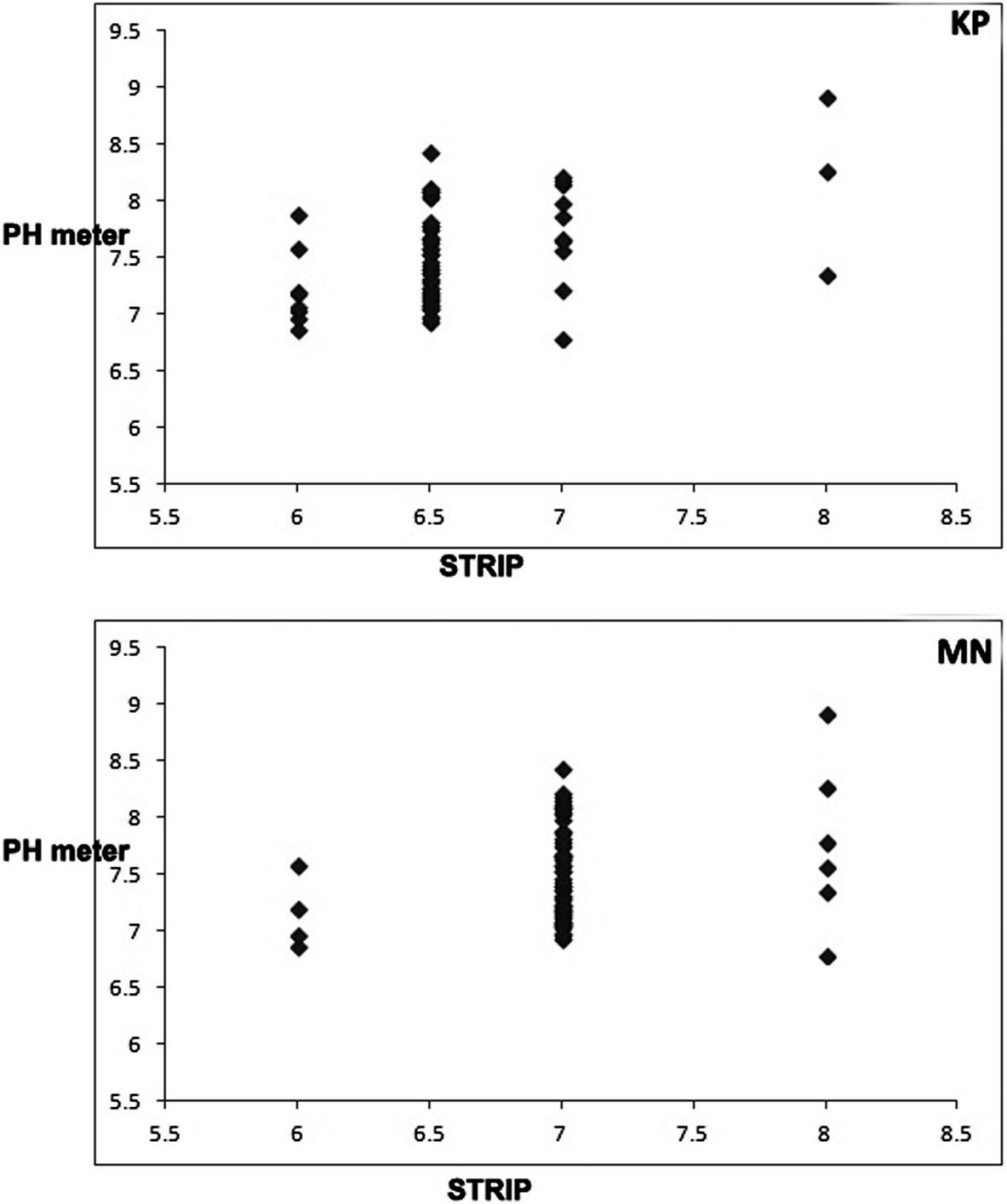
Scatterplot depicting the relationship between pH measured by human urinary dipsticks (x-axis) and by reference methods (y-axis). Upper and lower pictures indicate KP and MN human urinary dipsticks, respectively

The correlation results for SpG and pH between the two commercial dipsticks were good (*k* = 0.65) and moderate (*k* = 0.60), respectively (Table 1). Our findings revealed that there was also a poor correlation between the results of pH measured using the urine dipsticks and reference method (KP: r_S_ = 0.445, MN: r_s_ = 0.370, *P*s < 0.001) (Table 1) (Fig. 1). The mean ± SD values of the pH assayed using the KP, MN, and pH meter were 6.58 ± 0.41, 7.03 ± 0.39, and 7.70 ± 0.44, respectively. In general, it appears that human urinary dipsticks underestimate horse urine pH.

**Fig. 2:**
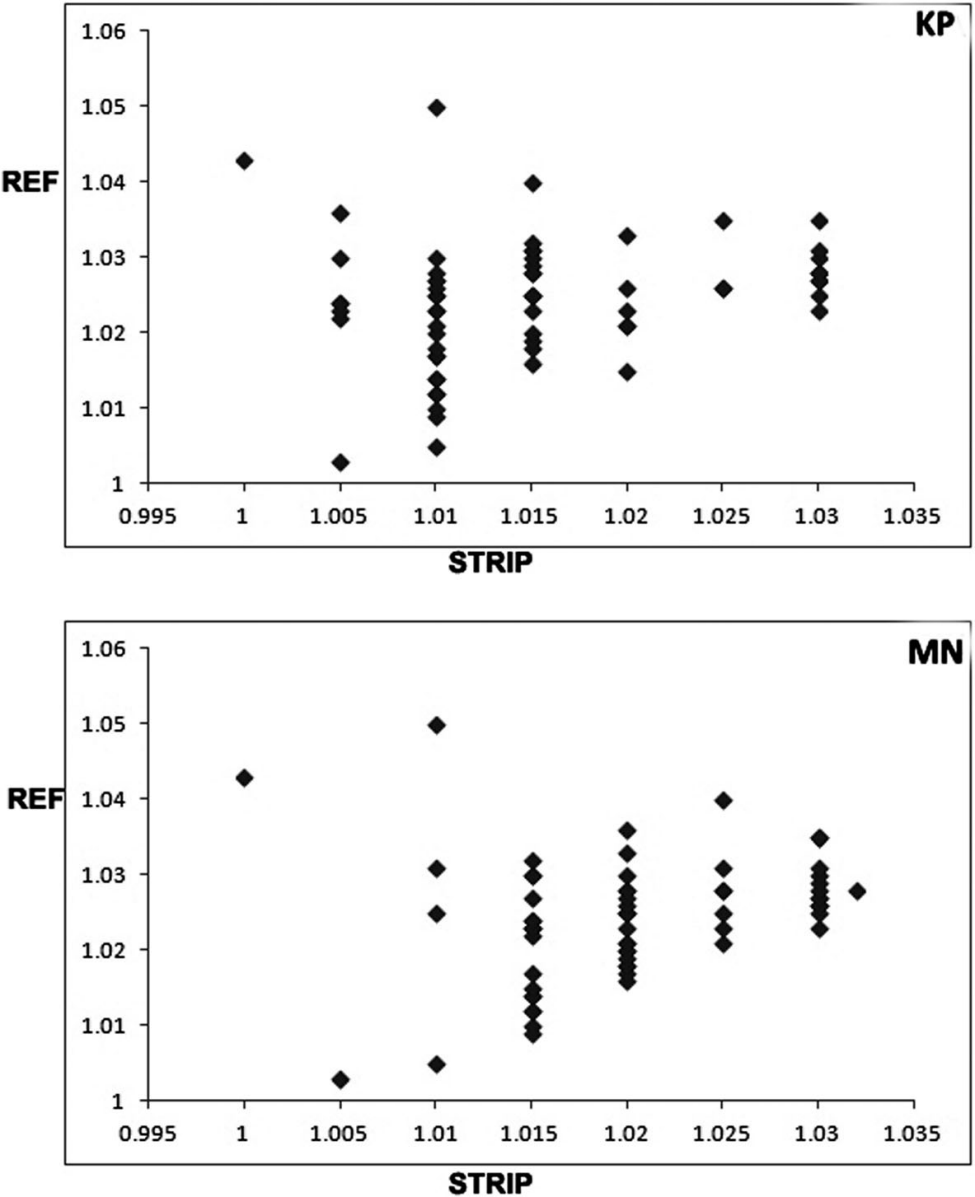
Scatterplot depicting the relationship between SpG measured by human urinary dipsticks (x-axis) and by reference methods (y-axis). Upper and lower pictures indicate KP and MN human urinary dipsticks, respectively

We also found similar findings regarding the measurement of urine SpG using the human dipsticks and related reference method in horses (KP: r_S_ = 0.285, MN: r_s_ = 0.338, *P*s < 0.001) (Table 1) (Fig. 2). Note that no significant difference was detected between SpGs measured before and after urine centrifugation (*P* = 0.64).

## Discussion

Findings of this study demonstrated that urinary dipsticks used for analysis of human urine are not reliable indicators of urine pH, SpG, or protein content in horses. In the current study, we detected many false positive protein results. A high number of false positive proteins was also reported in cattle, dogs, and cats [4, 5]. The investigation revealed that an alkaline urine pH was the possible cause for non-specific staining of the protein pad [5]. Similar to horses, previous studies indicated that human urine dipsticks had high sensitivity but low specificity for proteinuria in cattle, dogs, and cats [4]. Hence, these positive semiquantitative protein results should be interpreted with caution and confirmed by a reference method.

We found that the human urinary dipsticks underestimate horse urine pH. Given these problems, it is proposed that a portable pH meter be used for horses to achieve more accurate and robust results [6, 11]. Previous investigations suggested that human urinary dipsticks were reliable to measure urine pH in dogs [1, 4, 11], cats [4, 6], cattle [4, 10], and sheep [9]. There was a good to excellent correlation between urinary dipsticks and the reference method in cattle, dogs, and cats [4]. In comparison with horses, it was reported that urinary dipsticks overestimated pH in dogs [11]. The diagnostic performance of urinary dipsticks to estimate urine pH could be different in herbivores and carnivores, where herbivores and carnivores usually have an alkaline and acidic urine, respectively [9].

As noted in the results, the human urinary dipsticks could not reliably estimate urine SpG in horses. In general, human commercial urinary dipsticks do not show promising results to estimate SpG in other domestic animals. The correlation between the dipsticks and reference method was fair in cattle and dogs, but not in cats, in which the correlation was good [4]. In addition, in another study, a poor correlation was reported between SpGs as determined by the dipsticks and those determined using a refractometer in dogs [1]. Use of urinary dipsticks to measure SpG in humans is also controversial, where a clear disagreement emerged between the results of dipsticks and reference method [14].

There were some potential limitations in our study, where we couldn’t work on larger populations of horses or analyze abnormal urines obtained from horses presenting renal/urinary tract diseases. With inclusion of large number of normal and abnormal samples into the study, the efficacy of the urine dipsticks to detect cases with low SpGs (hyposthenuria) and proteinuria can be evaluated more reliably.

## Conclusion

In conclusion, we indicated that the human commercial urinary dipsticks were not reliable to correctly estimate urine protein, SpG, and pH in horses. However, some of these urinary parameters can be properly measured using human dipsticks in other domestic animals. Hence, as horse practitioners generally have access to human urinary dipsticks, we recommend that they confirm their results by a reference laboratory and use portable laboratory devises (such as a portable pH meter) [1, 6, 11]. Although use of specific veterinary urinary dipsticks can be another option, their diagnostic performance should be evaluated in further studies.

## Methods

### Animals and urinalysis

The current study was performed on 80 healthy adult horses from both genders (64 females and 14 males). The horses aged 6 years on average (range: 2–24 yrs. old) with an average body weight (BW) of 450 Kg (range: 400–500 Kg). The animals were kept in private barns and had ad libitum access to water. We obtained written informed consent from the horses’ owners to use the animals in our study. In this study, we only collected urine specimens and no further experiment was carried out.

The voided urine specimens (minimum volume of 10 cc) were collected and freshly (< 1.5 h) transferred to the laboratory and analyzed. Routine urinalysis was performed using two commercial human dipsticks (MN and KP).

First, the urines were checked for two physical properties (i.e., color and transparency) as a routine step of urinalysis procedure. If a sample had abnormal color (any color except yellow) or abnormal transparency, it would be excluded. None of the samples had abnormal color or transparency. After that, clinically relevant variables including pH, SpG, and protein were first measured semi-quantitatively using urine dipsticks and then assayed using the references methods. The urine dipsticks were read by two expert laboratory technicians, independently. For reference measurements, urine pH and SpG were quantitatively measured using pH meter (Metrohm, Switzerland) and handheld refractometer (ATAGO, Japan), respectively. The refractometer was calibrated daily with distilled water. In addition, for better accuracy, we measured urine SpG twice, i.e. before (whole urine) and after centrifugation (urine supernatant). Before pH measurement, the pH meter was calibrated using two buffers, including acidic (pH =4) and alkaline (pH = 7) buffers. The concentration of urine protein was determined using a standard colorimetric method (pyrogallol red) (Pars-Azmun, Iran) and clinical biochemistry analyzer (AUTOLAB, Ames, Rome, Italy). In pyrogallol red method, pyrogallol red-molybdate complex bound to basic amino acid groups of urine proteins with the resulting red colors quantified at a wavelength of 580 nm. In each run of the clinical biochemistry analyzer, internal control samples were used. The microscopic examination of unstained urine sediment was used to detect urine crystal and cast. Sediment was prepared from 7 ml urine by centrifugation (EBA8S, Hettich, Tuttlingen, Germany) at 1500 g for 5 min.

### Statistical analysis

The data was described as mean ± SD values for continuous variables and as proportions for categorical data. The correlation between the semiquantitative dipstick analysis and quantitative reference methods was determined using Spearman’s rank correlation coefficient. Correlations were graded based on the classification proposed by Papasouliotis et al. (2006) [15] (i.e., r_s_ = 0.93–0.100 as excellent, r_s_ = 0.80–0.92 as good, r_s_ = 0.59–0.79 as fair and r_s_ < 0.59 as poor correlation). The inter-rater agreement between the two observers or two dipsticks was concluded using Cohen’s kappa (*ĸ*) coefficient. The correlations were ranked based on the model proposed by Altman (1991) [16] (i.e., very good: *ĸ* = 0.81–1.00, good: *ĸ* = 0.61–0.80, moderate: *ĸ* = 0.41–0.60, fair: *ĸ* = 0.21–0.40, and poor: *ĸ* < 0.20). All statistical analyses were performed using SPSS.16 statistical package (USA, Chicago). A *P* value less than 0.05 was considered significant.

Additionally, the performance of human urinary dipsticks to detect positive protein samples in horses was calculated as sensitivity, specificity, positive predictive value (PPV), and negative predictive value (NPV). A concentration of 30 mg/dl was considered as the cut-off value.

## Data Availability

All data generated or analysed during this study are included in this published article.

## Abbreviations

BW: Body weight
KP: Kimia-Pajouhan
MN: Machery-Nagel
NPV: Negative predictive value
PPV: Positive predictive value
SpG: Specific gravity
